# Assessment of the impact of the Belgian and French food industry on obesity and population nutrition

**DOI:** 10.1093/eurpub/ckac130.100

**Published:** 2022-10-25

**Authors:** I Van Dam, O Allais, S Vandevijvere

**Affiliations:** 1 Lifestyle and Chronic Diseases, Sciensano, Anderlecht, Belgium; 2 Université Paris-Saclay, INRAE, UR ALISS, Ivry-sur-Seine, France

## Abstract

Major food industry players make commitments to improve food environments, but it remains unclear how well these commitments translate into practice. This study set out to quantitatively assess the nutrition-related commitments and practices, as well as the correlation between both, of major food companies in Belgium and France. The ‘Business Impact Assessment’ (BIA-Obesity) was applied to evaluate nutrition-related commitments and practices regarding product formulation, labelling, promotion and accessibility. Publicly available commitments were collected and companies given the opportunity to complete the information (2019-2020). Practices were evaluated applying following performance metrics: the proportion of products with Nutri-Score A or B, the percentage of products not-permitted to be marketed to children (World Health Organisation) and the proportion of ultra-processed food products (NOVA). Correlations between commitments and practices were calculated applying the Spearman's rank correlation coefficient. Overall BIA-Obesity scores for commitments were lower in France (median: 28%) than in Belgium (35%). Response rates in France (39%) were lower than in Belgium (56%). Median product portfolios in France contained less ultra-processed products (63%) and contained a higher proportion of products with a Nutri-Score A or B (38%) compared to Belgium (75% and 29%, respectively). In both countries a similar proportion of products was not-permitted to be marketed to children (81% in Belgium and 84% in France). Stronger company commitments did not translate into better performance metrics. Belgian food companies obtained a higher score for their nutrition-related commitments. French companies performed slightly better according to the performance metrics. In both countries there was ample room to improve commitments and practices. To improve food environments it is crucial to ensure that commitments are strengthened and translate into improved company practices.

**Key messages:**

- Food industry players make a range of nutrition and health related commitments, but these don’t necessarily result into better practices.
- The BIA-Obesity clearly identifies areas where food industry players can improve the comprehensiveness, specificity and transparency of their nutrition-related commitments and associated practices.

